# Infraeyebrow Blepharoplasty for Blepharochalasis of the Upper Eyelid: Its Indication and Priority

**DOI:** 10.1155/2012/975097

**Published:** 2012-01-15

**Authors:** Akira Sugamata

**Affiliations:** Department of Plastic and Reconstructive Surgery, Tokyo Medical University Hachioji Medical Center, 1163 Tatemachi, Hachioji, Tokyo 193-0998, Japan

## Abstract

Eyelid bags and blepharochalasis are the result of relaxation of lid structures like the skin, the orbicularis oculi, and mainly the orbital septum. Therefore, this aged appearance cannot be improved sufficiently with only a skin and orbicularis oculi resection. To improve this appearance, we use a very effective method of tucking of the orbital septum with infraeyebrow excision of the skin and the orbicularis oculi. Between January 2005 and April 2011, 103 patients (206 lids) were treated. There were 89 female and 14 male patients whose ages ranged from 43 to 75 years (mean = 65.2 years). After infraeyebrow excision, blepharoplasty with tucking of the orbital septum, the concealed lid crease becomes apparent and a good rejuvenation effect is obtained in all patients.

## 1. Introduction

Common early signs of aging in the upper eyelid include baggy appearance, blepharochalasis, and lateral drooping of the skin. These changes give the appearance of older eyes and are often associated with limitation of upper lateral visual fields. These aesthetic and functional complaints contribute to a patient's perception of the need for upper lid blepharoplasty. Infraeyebrow blepharoplasty has become widely accepted and performed in East Asia [1–6]. By using this operative technique, baggy appearance and blepharochalasis can be improved without extreme changes to a patient's facial appearance after the operation. This paper provides our functional indications for upper lid blepharoplasty.

## 2. Materials and Methods

Between January 2005 and April 2011, 103 patients (206 lids) were treated. There were 89 female and 14 male patients whose ages ranged from 43 to 75 years (mean = 65.2 years). The primary indication for our infraeyebrow blepharoplasty included patients who complained of aesthetic discontent and superior visual field limitation with blepharochalasis, but who did not have obvious levator muscle dysfunction. We indicated the operative procedure to patients whose levator function was better than 8 mm. They were followed up after 3 months to 6 years (mean = 10.6 months) (Table 1).

### 2.1. Design

Before the operation, we make a rough estimate to decide the quantity of skin to be resected by pinching the surplus skin with forceps in the infraeyebrow area while patients are in the sitting position.

**Figure 1:**
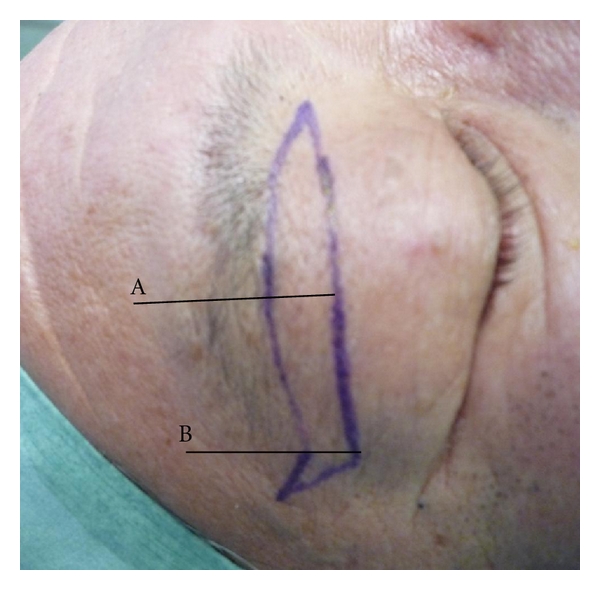
Design of the skin excision. (a) At the end point of the eyebrow, the excision line is extended straight up at an angle of 30°. (b) The greatest width is at the lateral two-thirds point of the eyebrow.

In our operative design, an upper excision line is drawn following the lower edge of the eyebrow from 2-3 mm lateral to the medial angle of the eye to the lateral end of the eyebrow. At the end point of the eyebrow, the excision line is extended upwards approximately 10–15 mm at an angle of 30°. The lower excision line begins from the same point as the upper line and increases in width laterally to the lateral two-thirds point of the eyebrow, thus creating a spindle shape. The lower line then extends almost parallel with the upper line to the axis through the end point of the eyebrow. At the cross-point of the lower excision line and the axis through the end point of the eyebrow, the lower excision line extends directly to the end point of the upper excision line (Figure 1). The greatest width of skin excised from the lateral eyelid to improve lid drooping should be 8–12 mm.

### 2.2. Operative Procedure

**Figure 2:**
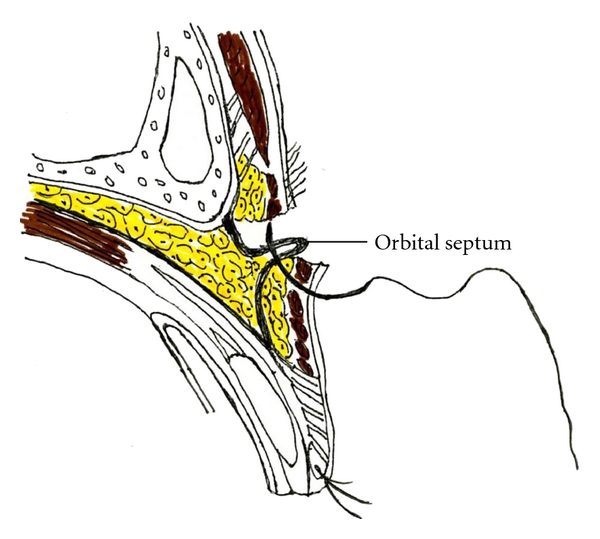
Tucking of the orbital septum.

In our operative procedure, we excise the skin and subcutaneous fat tissue totally from the surface of the orbicularis oculi muscle. After excision of the skin and fat tissue, we also excise the orbicularis oculi 1-2 mm inside the skin excision line and the preseptal fat tissue in the same area if it is distensible. Once this step is completed, the orbital septum is identified. The orbital septum is caught with forceps in this condition, and it should be pulled gently to confirm that it pulls the tarsal plate together. After confirmation of the orbital septum, the orbicularis oculi is sutured end to end with plication of the orbital septum using 5/0 nylon thread (Figure 2). Plication is always achieved with 5-6 stitches.

## 3. Results

**Figure 3:**
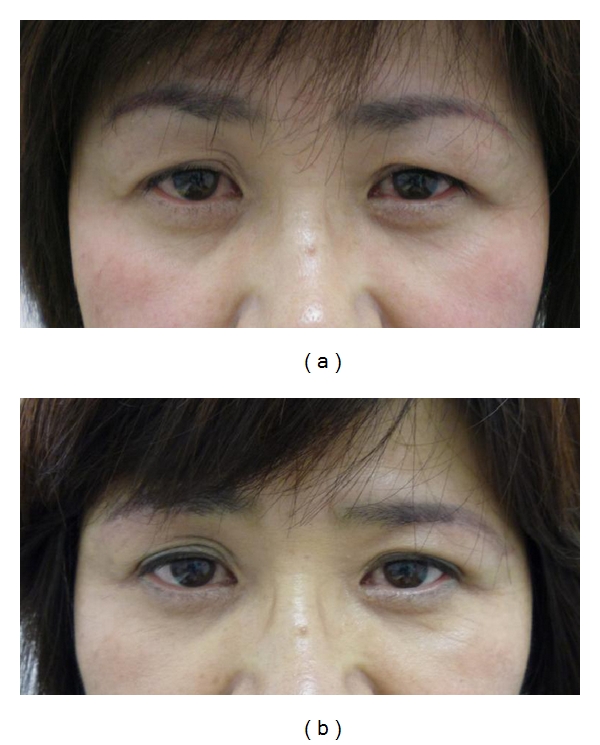
A 45-year-old woman. (a) Preoperative view, the greatest width of excised skin was 7 mm. (b) Six months after the operation.

**Figure 4:**
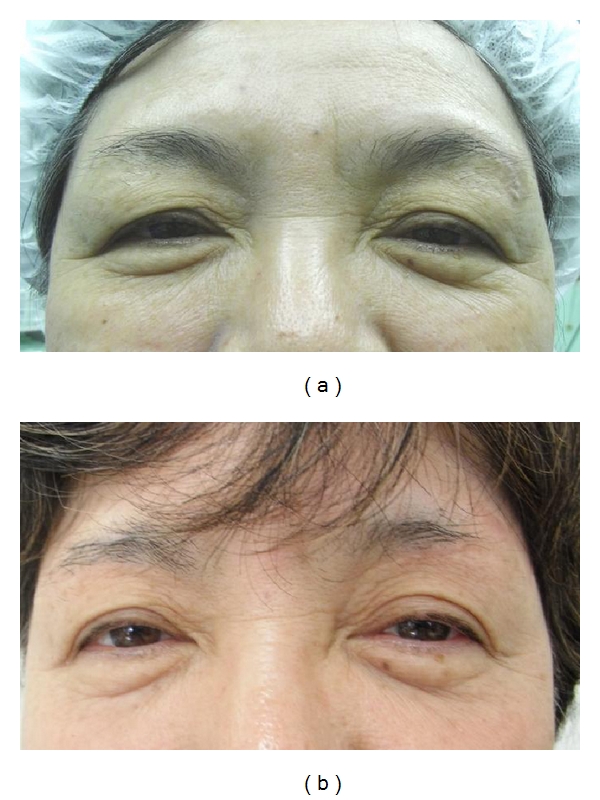
A 61-year-old woman. (a) Preoperative view, the greatest width of excised skin was 8 mm. (b) One year after the operation.

**Figure 5:**
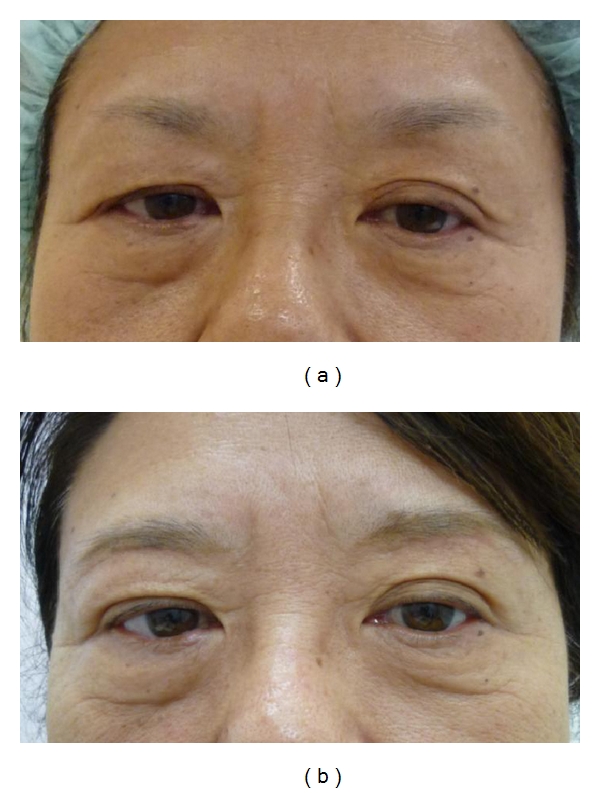
A 62-year-old woman. (a) Preoperative view, the greatest width of excised skin was 7 mm. (b) Seven months after the operation.

At the time of writing, the lateral drooping of the lid skin is improved and the lid crease becomes more clearly defined (Figures 3, 4, and 5). In addition, following surgery, the superior lateral visual field limitation is more refined than before the operation in all patients. The shortening ratio for the distance between the eyebrow and the upper cilia at the middle point of the upper eyelid in primary gaze is 8%–54.2% (mean = 28.4%). We have not encountered any serious complications. In some cases, there were complaints of sensory disturbance around the eyebrow, although this also resolved after several months. The scars are almost indistinguishable 6–8 months after the operation. There were two patients who underwent this procedure twice. One was an 85-year-old woman, who complained of a recurrence of blepharochalasis five years after the first surgery. The other was a 74-year-old woman who had left facial palsy caused by cerebral apoplexy, so she felt a slight insufficiency in the left side excision two years after the first operation. Both patients had the same procedure again.

## 4. Discussion

The most remarkable signs of the aging upper eyelid are drooping of anatomical structures like the skin, the orbicularis oculi muscle, and the orbital septum. Although upper eyelid skin thickness is not markedly affected by aging [7], the loss of elastic skin fibers and the development of skin laxity are both marked, and these changes parallel the advance of age [8]. Histologically, the whole muscle layer of the orbicularis oculi is also intact in aged eyelids, with no signs of aging such as loss of fibers, loss of adherence to surrounding structure, or ptosis. However, the skin and muscle, which are attached firmly together, both become attenuated; they have stretched together, and they have equal excess in aging [9]. The orbital septum is the most effective barrier against the anterior prolapse of the preaponeurotic orbital fat pads. The eyelid fat pads become prominent as the orbital septum thins and loosens with aging, especially in the lateral region, because the lateral element of the preaponeurotic orbital fat pads protrudes anteriorly under the inferior border of the lacrimal gland [10, 11]. According to these changes in ageing, to improve upper blepharochalasis, it is necessary to tighten these three elements.

Most upper eyelid blepharoplasty operations are performed through an incision into the lid crease (or low area in the upper lid) [12–18]. The greatest merit of these approaches is that correction of aponeurotic blepharoptosis can be performed at the same time without a separate procedure [13, 17]. In patients who request a double-eyelid upper blepharoplasty, a low incision in the upper lid is also available to create a new crease by fixing the pretarsal skin to the levator mechanism [14, 18]. Another benefit of these approaches is that the surgeon can regulate the height of the eyelid crease according to the wishes of the patient. However, from an aesthetic point of view, there are some shortcomings of lid crease and low lid incision in East Asian patients. As the width of the skin excision through the lid crease, or low lid in the increases, an unnatural appearance after the operation has often been reported [1, 3, 4]. One of these undesirable results is that a discrepancy between the nature and thickness of the sutured upper and lower skin in the upper lid accentuates the overhanging appearance of the upper skin on the crease line [1, 2]. Another undesirable result is that an overly defined lid crease after this operation often gives the patient's face a “surprised look” [1, 3, 4]. In patients who do not desire the double-eyelid procedure, retaining the natural East Asian flat eyelid without a lid crease is also difficult after a low incision in the upper lid [2, 4]. In addition, in these incisions, to remove lateral drooping skin from the upper eyelid, an extension of the skin excision must be carried out laterally beyond the lateral canthus, or to the corner of the eyebrow in an upward angle to prevent a lateral dog-ear of skin [19]. These lateral extended operative scars are often conspicuous in East Asian patients [3, 4]. To improve the shortcomings of blepharoplasty performed through the lid crease and low area in the upper lid, infraeyebrow blepharoplasty has been commonly accepted in East Asia [1–6]. To perform infraeyebrow excision, blepharochalasis can be improved without dramatic changes to the facial appearance after the operation. In addition, no conspicuous operative scars are produced in the lower lateral portion of the upper eyelid as a result of the operation and a more natural aesthetic appearance can therefore be obtained [1, 3, 4].

There are various operative designs in infraeyebrow blepharoplasty, and these depend on the surgeon undertaking the procedure. Hara et al. [5] indicated a spindle-shaped incision line, with the widest amount of resected skin being determined on the vertical line crossing the center of the pupil. We think that this design is not adequate because it will not improve lateral drooping of the skin sufficiently. Other authors [1–3] have described a marginal incision line of the eyebrow with an S-shaped lower incision line. This design makes it possible to excise the greatest width at the midpoint between the lateral canthus and the lateral limbus. However, we think the greatest width area of excision of the skin should be continued a little more laterally. In addition, the lateral extended incision is necessary to prevent a dog-ear in the skin because such lateral extended operative scars are often conspicuous. Our design enables us to excise the slack lateral eyelid skin and subcutaneous tissue sufficiently without any lateral extended operative scar.

Most of the reported cases of infraeyebrow blepharoplasty have only considered skin resection in the infraeyebrow area [2, 3, 5]. On the other hand, some authors have described resection of the skin and orbicularis oculi muscle [1, 6]. We already described that the most marked sign of upper eyelid aging is drooping of anatomical structures like the skin, the orbicularis muscle and the orbital septum, with the latter being the most effective barrier against anterior prolapse of the preaponeurotic orbital fat pads. Therefore, we should tighten these three elements in order to get the most effective result from upper blepharoplasty. Tucking of the orbital septum with infraeyebrow excision of the skin and the orbicularis oculi is very effective for improving baggy appearance and blepharochalasis. In addition, tucking of the orbital septum has the effect of mild static suspension of the tarsal plate due to septal attachment to the levator aponeurosis and the tarsal plate in the lower region of the upper eyelid. After our blepharoplasty procedure, superior lateral visual field limitation was also improved to a sufficient degree. We have not confirmed how long the benefits of plication of the orbital septum lasts, however, over the last 7 years there have been only two patients who complained of recurrence of blepharochalasis from 103 patients who underwent this technique.

The only aesthetic disadvantage of this approach is a slight brow flattening and shortening of the brow and ciliary distance [1, 4]. Actually, the brow and ciliary distance is noticeably shortened to a slight degree and brow flattening has been noted in some cases, but there have been no patients concerned by these outcomes.

Although our operative technique is useful in East Asian subjects, the indications of this technique in Caucasian subjects should be considered carefully [3, 4]. One reason is that the position of the eyebrow is low and the distance between the eyebrow and the cilia is short in Caucasian people, so it is difficult to excise a wide band of skin and muscle in the infraeyebrow area [3, 4]. The other reason is that most of the orbital septum is firmly attached to the levator aponeurosis in the upper eyelid in Caucasians [14, 20], so anatomically it is difficult to tuck up and suture the septum.

## Figures and Tables

**Table 1:** Characteristics of patients (*n* = 103).

	Number	Range (mean)
Sex		
Female	89	
Male	14	
Age		43–75 (65.2)
Follow-up period (months)		3–84 (10.6)
